# Adequate Vitamin D Intake Cannot Be Achieved within Carbon Emission Limits Unless Food Is Fortified: A Simulation Study

**DOI:** 10.3390/nu13020592

**Published:** 2021-02-11

**Authors:** Maaike J. Bruins, Ulla Létinois

**Affiliations:** DSM Nutrition Products, Wurmisweg 576, CH-4303 Kaiseraugst, Switzerland; ulla.letinois@DSM.com

**Keywords:** dietary modelling, sustainable diet, vitamin D intake, fortification, carbon emission

## Abstract

This study applied linear programming using a Dutch “model diet” to simulate the dietary shifts needed in order to optimize the intake of vitamin D and to minimize the carbon footprint, considering the popularity of the diet. Scenarios were modelled without and with additional fortified bread, milk, and oil as options in the diets. The baseline diet provided about one fifth of the adequate intake of vitamin D from natural food sources and voluntary vitamin D-fortified foods. Nevertheless, when optimizing this diet for vitamin D, these food sources together were insufficient to meet the adequate intake required, unless the carbon emission and calorie intake were increased almost 3-fold and 2-fold, respectively. When vitamin D-fortified bread, milk, and oil were added as options to the diet, along with increases in fish consumption, and decreases in sugar, snack, and cake consumption, adequate intakes for vitamin D and other nutrients could be met within the 2000 kcal limits, along with a relatively unchanged carbon footprint. Achieving vitamin D goals while reducing the carbon footprint by 10% was only possible when compromising on the popularity of the diet. Adding vitamin D to foods did not contribute to the total carbon emissions. The modelling study shows that it is impossible to obtain adequate vitamin D through realistic dietary shifts alone, unless more vitamin D-fortified foods are a necessary part of the diet.

## 1. Introduction

Food production has a considerable impact on greenhouse gas emissions [1]. The planet cannot sustain a continuation of the current dietary habits, especially when it comes to feeding the 10 billion people living on the planet by 2050. There is a growing understanding of the types of diets and food patterns that can be part of the solution in order to reduce environmental impact, while optimizing health in terms of nutrient adequacies when shifting dietary patterns [2]. Some governments have already incorporated sustainability into their national dietary guidelines [3]. Even though sustainability and health considerations are increasingly driving consumer purchasing decisions, consumers still face challenges when changing dietary habits in order to improve their nutrition and sustainability [4,5].

Vitamin D deficiency is among the most neglected major public health problems worldwide [6]. Surveys show that vitamin D deficiency is highly prevalent among all population groups, with severe deficiency (<25 nmol/L) and deficiency (<50 nmol/L) rates estimated to be 7% and 37% globally, respectively [6], and the vitamin D requirements are largely unmet in most populations [7,8]. In the Netherlands, one study found that no adults met the estimated average requirement for vitamin D [8]. Food provides a relatively small proportion of the vitamin D supply, while vitamin D produced in the skin from UVB light makes the greatest contribution [9]. An adequate intake of vitamin D-rich food is not the only difficulty, as adequate sunlight exposure can be a challenge with sun avoidance and less time spent outdoors. The high prevalence (83%) of low serum 25(OH)D levels <50 nmol/L in Dutch adults suggests that vitamin D from diet and UVB exposure combined are not adequate [10]. To ensure that individuals consume adequate vitamin D, irrespective of their exposure to sunlight, the Institute of Medicine (IOM) and the European Food Safety Authority (EFSA) set the adequate intake for vitamin D based on assumed low sun exposure and the intake needed in order to achieve a serum 25(OH)D of ≥50 nmol/L, a level unlikely to pose adverse musculoskeletal health outcomes [11,12]. Moreover, experts have highlighted the potential immunomodulant, anti-inflammatory, and anti-infective roles of vitamin D beyond bone and muscle health [9,13].

However, obtaining an adequate intake of vitamin D from the diet alone is difficult, as only few foods naturally contain significant amounts of vitamin D [14]. As vitamin D food sources include mainly oily fish, meat, dairy, and eggs, shifting to more plant-based diets is likely to further aggravate the risk of vitamin D deficiency. It remains controversial among professionals whether sufficient vitamin D can be obtained from a healthy diet. This study simulated the shifts needed within a Dutch “model diet” to overcome vitamin D shortfalls, as well as the consequences for calorie intake and carbon emissions. In addition, dietary shifts were modelled by extending the diet with fortified milk, bread, and vegetable oils optimizing for vitamin D, as well as the vitamin D and carbon footprint combined.

## 2. Materials and Methods

### 2.1. Linear Modelling Methods

The linear modelling program Optimeal^®^ 2.0 (Blonk Consultants, Gouda, the Netherlands) was used to model scenarios of dietary shifts in the Netherlands. The program can propose dietary shifts from the current diet based on nutritional or environmental dietary goals, optimizing for popularity, by searching for scenarios of foods that resemble current diets as closely as possible. The program can set boundaries (constraints) for 36 nutrients, and for energy to be fulfilled or limited through the upper boundaries. In all scenarios, the recommended nutrient intake (RNI) and adequate intake (AI) were set as the lower boundaries, and tolerable upper intake level (UL) and maximum reference value (MRV) were set as the upper boundaries. We combined the data from the Dutch National Food Survey and Food Composition Database and the Life Cycle Assessment databases, resulting in a consolidated dataset with daily amounts consumed, nutritional value, popularity estimates, and carbon footprint estimates for 251 food items. The intake frequency of food items were used as a proxy for food popularity. This Dutch model diet was optimized for vitamin D (and carbon footprint) through linear modelling using the following scenarios.

### 2.2. Scenario 1: Optimizing the Current Diet for Vitamin D without Energy Constraints

In the first scenario, the baseline diet was optimized for an adequate intake of vitamin D. The baseline diet included some voluntary vitamin D-fortified foods, such as juices, fat spreads, soy-drinks, and breakfast cereals. The recommendations for nutrients had to be fulfilled, while the upper limits for calories were removed to allow the optimized diet to reach the adequate intake of vitamin D (13.4 µg/d).

### 2.3. Scenario 2: Optimizing the Current Diet for Vitamin D within Energy Constraints

In the second scenario, the diet was optimized for vitamin D, limiting energy intake to 2000 kcal and fulfilling nutrient recommendations. The diet was optimized to reach 9.6 µg/d, the maximum achievable amount of vitamin D, within a 2000 kcal constraint.

### 2.4. Scenario 3: Optimizing the Current Diet with Additional Fortified Foods for Vitamin D

In this scenario, vitamin D-fortified whole grain breads, semi-skimmed milk, and oil (soy, arachidic, and sunflower) were added to the food repertoire. These diets were optimized for a vitamin D intake of 13.4 µg/d, meeting nutrient recommendations, within a 2000 kcal limit. Combinations of two or three vitamin D-fortified foods were modelled.

### 2.5. Scenario 4: Optimizing the Current Diet with Additional Fortified Foods for Vitamin D and CO_2_

Like the previous scenario, vitamin D-fortified bread, milk, and oil were added to the diet. Both an adequate vitamin D intake and carbon footprint were set as the goals, while fulfilling nutrient references within a 2000 kcal limit. Either a capped (limit at 3.9 kg CO_2_ eq) or a 10% reduced footprint (limit at 3.5 kg CO_2_ eq) was simulated.

### 2.6. Food and Nutrition Data Used in the Model

Chronic food consumption (food records taking into account different survey periods) from the 24- h dietary recall Dutch National Food Consumption Survey (DNFCS) of 2003 was retrieved from the EFSA Comprehensive European Food Consumption Database [15] at food classification system “FoodEx” level 3, i.e., food category sub-items, such as type of cheese. The nutritional composition of foods was defined using the Dutch Food Composition Database (NEVO) food composition tables of 2016 [16]. If the food item was not available in the NEVO database, the nutritional profile was selected from the United States Department of Agriculture (USDA) food composition database. After adding hypothetical fortified foods (see Section 2.8), this resulted in a set of 251 food items.

The nutrition goals were based on the RNI’s and AI’s for vitamins and minerals, as recommended by the EFSA for adults [17]. The adequate intake for vitamin D is based on minimal exposure to sunlight [11]. For modelling purposes, reference values were averaged for men and women. Assuming an average 2243 kcal consumption for adult women and men at a moderate physical activity level [18], the dietary reference values were also adjusted to 2000 kcal. For instance, the vitamin D reference value of 15 µg/d was adjusted to 13.4 µg/d per 2000 kcal (Table A1). The upper bounds or maximum reference values (MRVs) for carbohydrates, free sugars, total fat, saturated fatty acids, trans-fatty acids, cholesterol, and sodium were based on reference values from the World Health Organization and the Food and Agriculture Organization [19] (Table A1).

### 2.7. Nutrient Density of the Diet

The baseline and modelled diets were standardized to 2000 kcal in order to calculate the nutrient density per 2000 kcal diet, which allowed for comparisons between countries or genders, irrespective of calorie intake or reporting, providing a good reflection of the diet quality. We calculated the mean adequacy ratio (MAR) as an overall measure of the nutrient adequacy of the diet. The MAR was calculated as desired nutrients in a 2000 kcal diet as a percent of the RNI or AI, truncated at 100%, and averaged for 26 qualifying nutrients [20]. The mean excess ratio (MER) was calculated as percent of the MRV, and was averaged for 6 undesired nutrients (total fats, saturated fatty acids, trans fatty acids, cholesterol, added sugars, and sodium) [20]. The added sugars were estimated from the sum of the food categories, in which mono- and di-saccharides almost exclusively represented the added sugars, and by subtracting the estimated lactose content in dairy foods from the total mono- and di-saccharides.

### 2.8. Fortified Foods Used in the Model

The current diet in the Netherlands already includes some vitamin D-fortified products, such as breakfast and porridge cereals, on average fortified at 4.2 µg/100 g and 16.5 µg/100 g, respectively, as well as fat spreads, fortified on average at 7.5 µg/100 g of vitamin D. Bread and vegetable oil offer a suitable opportunity for improving vitamin D intake through fortification, as they are consumed by a large proportion of the population in fairly constant amounts [21], are among the categories considered acceptable by consumers in Nordic countries [22], and their fortification with vitamin D is technically feasible. Fortified milk offers another option to voluntary fortify food, but this may reach less people, as some population groups do not consume milk. Vitamin D levels of 2, 6, and 15 µg/100 g in semi-skimmed milk, whole grain bread, and vegetable oil, respectively, were selected. Fortified bread and milk were constrained to two servings daily, so as to avoid proposing an unrealistically high consumption of these food items in the simulated diet.

### 2.9. Carbon Footprint Data Used in the Model

Optimeal^®^ 2.0 contains the environmental impact data of more than 200 food products, including carbon footprints. If the carbon footprint data were not available, they were obtained from the Agri-footprint^®^ 3.0 Life Cycle Inventory food database (SimaPro Life Cycle Analysis software 8, Amersfoort, the Netherlands). The implementation of the impact assessment methods in SimaPro were used without modification. The carbon footprint was calculated using the IPCC 2013 GWP 100a assessment method and the results were expressed as kg CO_2_ equivalents, using the associated characterization factors for the relevant greenhouse gases. This modelling study uses an attributional life cycle assessment estimating what share of the global environmental burdens belongs to a product. It was estimated that one kilogram of vitamin D3 can have a carbon footprint of less than 200 kg CO_2_ equivalent based on primary data for the production of vitamin D3 (internal data). The carbon footprints of the whole grain bread, semi-skimmed milk, and oil were about 0.09, 0.12 kg, and 0.2–0.4 CO_2_ equivalent per 100 g, respectively. Adding 2, 6, and 15 µg of vitamin D per 100 g of bread, milk, and oil, respectively, added ~0.001% CO_2_ to the total CO_2_ footprint of the food product.

## 3. Results

### 3.1. Scenario 1: Optimizing the Current Diet for Vitamin D without Energy Constraints

**Figure 1:**
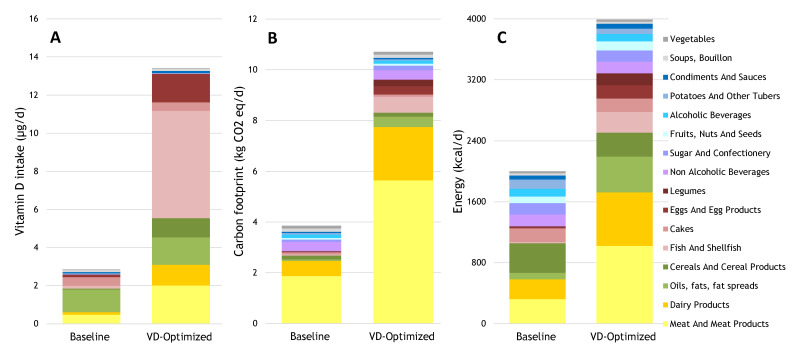
Daily contributions to (**A**) vitamin D intake, (**B**) carbon footprint, and (**C**) energy intake from the baseline diet and the diet optimized for vitamin D assuming no energy intake restrictions.

Fish; meat products; dairy; eggs; and some voluntary fortified foods such as juice, fat spreads, and breakfast cereals, are the main sources of vitamin D in the Dutch diet (Figure 1A). The baseline diet provided about 3 µg/d of vitamin D per 2000 kcal, contributing 21% of the adequate intake of vitamin D per 2000 kcal. Animal-source products provided 2 µg/d of vitamin D, fortified foods provided 1 µg/d, and mushrooms provided 0.01 µg/d. Achieving 13.4 µg/d of vitamin D was not possible with the current diet within the 2000 kcal intake limit. Therefore, the upper constraints for energy intake were removed. An adequate vitamin D intake could be reached when increasing the carbon footprint 2.8-fold (Figure 1B) and increasing the calorie consumption two-fold (Figure 1C). The increase in the carbon footprint of the optimized diet compared with the baseline diet was mainly attributable to an increase in the carbon footprints of meat products (3-fold); dairy (4-fold); oils, fat, and fat spreads (7-fold); egg products (11-fold); fish (15-fold); and legumes (17-fold), respectively (Figure 1B).

### 3.2. Scenario 2: Optimizing the Current Diet for Vitamin D within Energy Constraints

**Figure 2:**
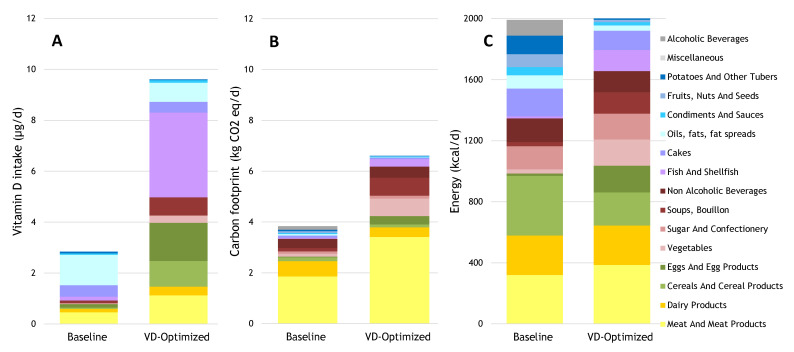
Daily contributions to (**A**) vitamin D intake, (**B**) carbon footprint, and (**C**) energy intake from the baseline diet and the diet optimized for vitamin D within a 2000 kcal boundary.

Despite the inclusion of voluntary vitamin D-fortified foods in the Dutch baseline diet, only 9.6 µg/d instead of the adequate 13.4 µg/d vitamin D could be achieved within the energy constraints of 2000 kcal (Figure 2A). To achieve 9.6 µg/d of vitamin D, fish (smoked herring and eel, and fish fingers), egg products (fried and boiled eggs), meat products (minced meat balls, meat soup, pate, and lean sausages), fortified breakfast cereals (cornflakes), fortified margarine, butter cakes, and vegetables (fried mushrooms) provided most of the vitamin D in the vitamin D-optimized diet (Figure 2B). Optimizing the diet for vitamin D within the 2000 kcal boundary increased the carbon footprint 1.7-fold compared with the baseline diet (Figure 2B). The carbon footprint increased 11-fold for egg products, 7-fold for fish, 6-fold for vegetables, and 2-fold for meat products, relative to the baseline. To achieve the vitamin D goals while meeting the nutrient recommendations, calorie consumption from egg products, fish, vegetables, and meat products would need to increase 11-, 10-, 6-, and 2-fold, respectively, while reducing calories from most other food categories (Figure 2C).

### 3.3. Scenario 3: Optimizing the Current Diet with Additional Fortified Foods for Vitamin D

**Figure 3:**
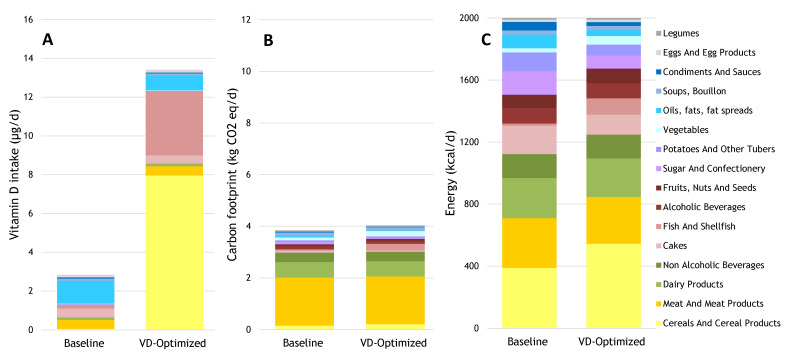
Daily contributions to (**A**) vitamin D intake; (**B**) carbon footprint; and (**C**) energy intake from the baseline diet and diet with additional fortified bread, milk, and oil optimized for vitamin D within a 2000 kcal boundary.

When adding vitamin D-fortified bread, milk, and oil to the Dutch baseline diet, it was possible to optimize the diet with an adequate vitamin D intake of 13.4 µg/d, meeting the other nutrient requirements while remaining within the 2000 kcal consumption constraint (Figure 3A). Vitamin D from fish increased 22-fold from baseline, and from fortified bread and breakfast cereals it increased 170-fold from baseline (Figure 3A). Fortified bread was proposed over fortified milk or oil as source of vitamin D. When fortified bread was excluded as a dietary option, fortified oil was proposed over fortified milk (data not shown). When fortified oil was also excluded, fortified milk could fulfill the vitamin D requirements adequately (data not shown). Optimizing the baseline diet to meet the adequate intake of vitamin D involved an 8% increase in the total diet carbon footprint, coming mostly from fish and vegetables, of which the carbon footprints increased 6- and 2-fold compared with baseline, respectively (Figure 3B). To achieve vitamin D goals while also meeting the other nutrient recommendations, calorie consumption from fish and vegetables would need to increase 8- and 2-fold, respectively (Figure 3C). In exchange, calories from cakes, sugar, snacks, potatoes, and tubers would need to decrease.

### 3.4. Scenario 4: Optimizing the Current Diet with Additional Fortified Foods for Vitamin D and CO_2_

**Figure 4:**
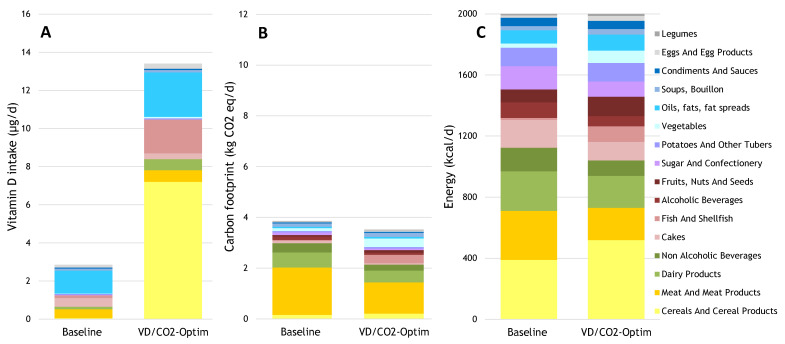
Daily contributions to (**A**) vitamin D intake; (**B**) carbon footprint; and (**C**) energy intake from the baseline diet and the diet with additional fortified bread, milk, and oil optimized for vitamin D and CO_2_ within a 2000 kcal boundary.

Optimizing the diet for an adequate vitamin D intake and capped CO_2_ emission, while satisfying nutrient recommendations, was feasible through a small shift from animal-source foods to fortified cereals (data not shown). A 10% CO_2_ footprint reduction could only be achieved when removing the minimum nutrient recommendations or significantly shifting to less popular food items. In the latter scenario, vitamin D was obtained from an increased consumption of fish and fortified bread and breakfast cereals (Figure 4A), and from a shift from unfortified to fortified foods. The net 10% reduction in CO_2_ was a result of less CO_2_ (−33%) from meat, dairy, non-alcoholic drinks, cakes, sugar, and snacks, and a smaller increase in CO_2_ from the total of legumes, fruits, nuts, seeds, fish, and vegetables (Figure 4B). Meeting the vitamin D and CO_2_ goals while the satisfying nutrient recommendations required a significant shift in calorie intake (−33%), moving from meat, dairy, non-alcoholic drinks, cakes, sugar, and snacks, towards eggs (2-fold), vegetables (3-fold), and fish (8-fold; Figure 4C).

### 3.5. Nutrient Density of the Current Diet and the Optimized Diets

The calculated MAR and MER of the usual and optimized diet are shown in Table 1. The MAR of the desired nutrients in the Dutch baseline diet was 86%, with vitamin D being the first limiting nutrient (followed by seafood omega-3 fatty acids and fiber). The MER of the nutrients overconsumed relative to the maximum reference values was 120% (20% excess). Optimizing the diet for vitamin D and satisfying the nutrient recommendations without energy constraints increased the MAR to 100%, but increased the MER 2.4-fold. After adding vitamin D-fortified whole grain bread, milk, and oil, the MAR increased to 100% and the MER decreased to 112%. Setting additional goals to reduce CO_2_ by 10% by compromising on popularity reduced the MER to 100%

## 4. Discussion

This simulation study demonstrates that even with a diet that is relatively abundant in vitamin D-rich foods, it is not possible to achieve an adequate intake of vitamin D without greatly increasing the carbon emission and calorie intake. Adding vitamin D-fortified options to the diet allowed for achieving the adequate intake of vitamin D and nutrient recommendations without sacrificing the carbon footprint and popularity of the diet.

The adequate vitamin D intake of 15 µg/d set by the EFSA and IOM represents the average adequate intake to achieve a serum 25(OH)D of ≥50 nmol/L [11,12]. The assumed low average year-round sun exposure in these dietary guidelines is realistic for the northern latitude of the Netherlands, with a high prevalence of vitamin D deficiency [10]. The Dutch model diet contributed approximately 3 µg/d per 2000 kcal (i.e., 20% of the adequate intake for vitamin D). This is comparable to the average vitamin D intake of 4.1 µg/d reported for European countries [23]. Two-thirds of the vitamin D in the Dutch model diet came from animal-source foods, one-third from voluntary vitamin D-fortified foods, and mushrooms contributed marginally.

In this study, the Dutch model diet was optimized to meet the adequate intake for vitamin D. This was only achievable when the calorie intake increased 2-fold and the carbon footprint increased almost 3-fold. However, the inclusion of additional vitamin D-fortified bread, milk, and oil in the diet, along with shifts in energy consumption towards fish and more plant-based nutrient-dense food sources, allowed for achieving an adequate vitamin D intake with minor compromises on the carbon emission and popularity of the diet within 2000 kcal limits. Clearly, the improvement in vitamin D adequacy (from 21% to 100%) and average nutrient adequacy (from 86% to 100%) was larger than the 8% increase in the carbon footprint. As only µg amounts of vitamin D are added to foods, vitamin D contributes only 1 permille to the carbon footprint of a food product and not to the total diet. A 10% reduction in carbon emissions while meeting the nutrient recommendations was feasible when shifting the intake of popular products such as meat, dairy, sugar, snacks, cakes, and non-alcoholic drinks more towards fish, fruits, nuts, vegetables, and eggs. However, these dietary changes may be less acceptable. Large reductions in meat, fish, eggs, and dairy products are not an option, as they provide essential or important sources of calcium; iodine; zinc; iron; and vitamins B2, B3, B5, B6, B12, and D [24,25,26].

Our study has various limitations; first, the food survey used in the model was from 2003, whereas food patterns likely changed over recent years. Second, the study focused solely on vitamin D intake relative to dietary references. Future work could consider integrating sun exposure as a source of vitamin D status in the model. Third, only the carbon footprint was selected as indicator of environmental impact, but other aspects such as land occupation and water use were not considered. The main strength of the study was the integral consideration of the popularity, nutrition, and climate aspects of the diets. Additional drivers of dietary choices, such as price, could be addressed in future research.

Previous studies concluded that without the universal fortification of staple foods or a dramatic increase in fish consumption, the current vitamin D intakes are too low to meet the recommendations or to sustain a healthy vitamin D status in the population [27,28,29,30]. This is substantiated by our findings, showing that an unrealistic increase in animal-source foods and the consequent carbon footprint is needed in order to meet the adequate vitamin D intake. Fortified whole grain bread was proposed over other fortified foods as a source of vitamin D, probably because it contributes to filling the fiber intake gap in the Netherlands, is popular, and has a relatively favorable carbon footprint. When fortifying foods, acceptable foods with a low carbon footprint addressing a nutrient gap should be considered.

Achieving sufficient vitamin D from the sun has become an increasing challenge, with more sun avoidance, time spent indoors, and a narrowing gap between beneficial and harmful UV exposure time to obtain desirable vitamin D. Various simulation studies show that the inclusion of vitamin D-fortified foods in the diet can be a viable and safe approach to improve intakes or reduce the prevalence of inadequate intakes [30,31]. Vitamin D-fortified bread and milk were able to reduce low vitamin D status in the winter season [32]. Food fortification with vitamin D in order to improve public health has been shown to be a cost-effective approach [33]. In voluntary fortification approaches, it is important that it is well-accepted by the population itself [33]. Consumers’ perceived health benefits and the appropriateness of the product are important drivers of purchasing and consumption [22]. In Finland, voluntary vitamin D fortification of milk products and fat spreads has been well-accepted since 2003, and helped the Finnish population reach vitamin D levels ≥50 nmol/L in 2011 [34]. Enriching the vitamin D content of eggs, milk, and meat by adding vitamin D to feed represents another potential complementary approach to address inadequate vitamin D intake at a population level [35]. Animal-source foods continue to be an important part of diets, as they provide micronutrients that are difficult to obtain in adequate quantities from plant-source foods alone. Vitamin D supplement intakes and recommendations have also shown to contribute significantly to achieving sufficient vitamin D status [34,36].

The present study shows that adequate intakes for vitamin D cannot be achieved with the current diet alone within realistic calorie and carbon emission limits, and additional vitamin D sources are needed to overcome the shortfalls. Universal fortification along with small dietary shifts represents an approach to improve the vitamin D status of the general population, at a high acceptability without affecting the carbon footprint.

## Figures and Tables

**Table 1:** The mean adequacy ratio (MAR) and mean excess ratio (MER) of the Dutch diet: (1) baseline, (2) after the inclusion of fortified milk and bread, and (3) optimizing for vitamin D and (4) vitamin D and CO_2_.

	Current	Scenario 1 Usual Diet Vitamin D Goals No Energy Limits	Scenario 2 Usual Diet Maximum Vitamin D 2000 kcal Limits	Scenario 3 Extra Fortified Foods Vitamin D Goals 2000 kcal Limits	Scenario 4 Extra Fortified Foods Vitamin D Goals CO_2_ Goals 2000 kcal Limits
Mean adequacy ratio (MAR)	86%	100%	100%	100%	100%
Mean excess ratio (MER)	120%	242%	154%	112%	100%

## Appendix A

**Table A1:** Dietary reference values averaged for men and women adjusted for 2000 kcal energy consumption applied in the dietary modelling.

		RNI ^1^ or AI ^2^	UL ^3^ or MRV ^4^
Energy	kcal	2000	2000
Protein ^5^	g	58.1	125
Polyunsaturated fatty acids	g	13.3	26.6
Linoleic acid	g	8.9	19.4
α-Linolenic acid	g	0.9	4.8
Fiber	g	25	
Water	g	2300	3800
Alcohol	g	0	10
DHA+EPA ^6^	mg	250	1000
Vitamin A	µg RAE	624	3000
Thiamin (B1)	mg	0.84	
Riboflavin (B2)	mg	1.43	
Niacin (B3)	mg NE	13.4	
Vitamin B6	mg	1.52	25
Folate (B9)	µg FE	294	1000
Vitamin B12	µg	3.57	
Vitamin C	mg	91	
Vitamin D	µg	13.4	100
Vitamin E	mg	10.7	300
Vitamin K	µg	62	
Calcium	mg	847	2500
Copper	mg	1.3	5
Iodine	µg	156	600
Iron	mg	10.9	70
Magnesium	mg	290	530
Phosphorus	mg	490	3000
Potassium	mg	3121	
Selenium	µg	62	300
Zinc	mg	10.2	25
Tryptophan	g	0.3	
Threonine	g	1.1	
Isoleucine	g	1.5	
Leucine	g	3.0	
Lysine	g	2.3	
Methionine	g	0.8	
Valine	g	2.0	
Histidine	g	0.8	
Carbohydrates	g		300
Added sugar	g		50
Total fat	g		78
Saturated fatty acids	g		22
Trans-fatty acids	g		2.2
Cholesterol	mg		300
Sodium	mg		2000

^1^ Recommended nutrient intake (RNI); ^2^ adequate intake (AI); ^3^ tolerable upper intake level (UL); ^4^ maximum reference value (MRV); ^5^ at body weight of 70 kg; ^6^ DHA—docosahexaenoic acid; EPA—eicosapentaenoic acid.
